# Remarks on the Surgery of the Kidney

**Published:** 1885-12

**Authors:** Lawson Tait


### Remarks on the Surgery of the Kidney.

By Lawson Tait, F. R. C. S.

In a communication to the Canada *Lancet*, Mr. Lawson Tait gives his notes of some cases of kidney disease, calling for operation, recently met with; among these, a case of movable kidney, which we give, with the concluding remarks of the paper:

J. W., æt. 45, had a large painful tumor on the right side, slightly to the right side, but mostly lying over the vertebral column; it clearly fluctuated, and was freely movable. It was diagnosed as a cyst of the mesentery. I opened the abdomen, for the purpose of removing it, on the 18th of June, and at once came upon a thin-walled cyst containing about three pints of clear fluid, and, after emptying it, I endeavored to shell it out from the folds of the mesentery under which it appeared to lie. This I succeeded in doing, until I came upon a fresh substance, which spe dily pronounced itself to be the kidney. I had, in fact, been dealing with a case of hydronephrosis, in which the pelvis of the kidney had become so completely dilated that the body of the organ itself lay quite behind. Of course, I had to complete the operation by removing the kidney itself. Only one ounce of urine was passed on the day of the operation, but the quantity steadily rose until it was thirty ounces, when she left for home on July 4th. She has since remained in good health, and the wound is perfectly healed.

Remarks.—The surgery of the kidney has now advanced to such a stage, that we may speak pretty positively of what can and what ought to be done in all cases of tumors of this organ. What I have to say now is very much a repetition of what I have said in previous papers on this subject, with the exception that I have had, as I have already indicated, to modify somewhat my belief concerning movable kidneys; but even here I can confirm much of what I have already said on the question. This abnormality is so rare that this is the first case I have ever seen, in a practice which now extends over twenty-five years, and which includes forty operations upon the kidney, and more than twelve hundred abdominals. The second point deserving notice is, that all of these forty operations, with one exception, have been performed on the right kidney, a circumstance which certainly is very remarkable, and must be something more than a mere coincidence. Some months ago, in a communication made to the Obstetrical Society, I told the story—now some fifteen years old—of an interview I had with a board of examiners, whom I could not satisfy concerning the treatment to which I would subject a patient submitted to me for the purpose of the examination, and who was burdened with a large suppurating tumor of the kidney. I told the board that I should remove the kidney, as everything else seemed to have been tried fruitlessly. In reply, they told me that my surgical enthusiasm seemed to be greater than my knowledge of the practice of medicine, and we parted, never to resume further association. A singular change has occurred since then, for the kidney promises to be one of the most brilliant fields for the achievements of new surgery. Out of my forty operations on diseased kidneys, including abscesses, hydatids, sarcomas, and stones in the pelvis, I not only had thirty-eight recoveries, but I have had—so far as I know up to the time of writing—complete cures in thirty-eight out of the forty operations. In the fortieth case I failed, because I did too much. I removed a kidney with a large number of chronic abscesses in it, when I ought simply to have opened it and drained it, as an expedient preparatory to its subsequent removal. This patient died of shock, and in this I learnt the lesson which I shall always follow in future in such cases, of opening the kidney, in order to ascertain its condition exactly before I remove it. I really think that, in this conclusion, I have to sum up all my experience in renal surgery.

There can be no question that interference with the large malignant tumors, which we see in children under fourteen and fifteen years of age, is simply nonsense. Even if the little sufferers recover from the terrible operation which is performed in such a case, the disease will recur, and no good will have been done. But in all other tumors of the kidney, if the patient is sufficiently ill to justify interference, the disease may be attacked with a perfect certainty of procuring relief in every case, and complete cure in probably 90 per cent. I do not think it matters much whether the organ be attacked by what is called the abdominal method, or the lumbar, as far as the immediate success of the operation is concerned. But I have a strong preference in my own practice for the abdominal method, if there is any likelihood of its being decided to remove the kidney, for, in this way, the condition of the other organ may be ascertained before the diseased one is removed. If, however, it has been determined to perform nephrotomy, and the removal of the diseased organ is a question which has been dismissed, then, I think, the lumbar incision may be the preferable of the two. In such a case, the operation of nephrotomy is an extremely easy one, and even the removal of the kidney is not a proceeding accompanied by much danger or difficulty, unless it has been too long delayed.

